# Prevalence of hypertension in overweight and obese children from a large school-based population in Shanghai, China

**DOI:** 10.1186/1471-2458-13-24

**Published:** 2013-01-11

**Authors:** Xi Lu, Peng Shi, Chun-Yan Luo, Yue-Fang Zhou, Hui-Ting Yu, Chang-Yi Guo, Fan Wu

**Affiliations:** 1 Shanghai Municipal Center for Disease Control and Prevention & Shanghai Institutes of Preventive Medicine, 1380 W. Zhongshan Road, Shanghai, 200336, China; 2 Department of Clinical Epidemiology, Children’s Hospital of Fudan University, Shanghai, 201102, China

**Keywords:** Obesity, Overweight, Hypertension, Children and adolescents, China

## Abstract

**Background:**

The ongoing rise in the prevalence of hypertension in children and adolescents is considered to be accompanied with the epidemic of childhood overweight and obesity. In this study, we established a large scale cross-sectional study in Shanghai, China, which presented a new evidence for the correlation of hypertension prevalence with overweight and obesity stages in Chinese children and adolescents.

**Methods:**

A school-based cross-sectional study was conducted during February to December 2009 in Shanghai, China, including total 78,114 children and adolescents. Body weight, height, waist circumference (WC) and blood pressure (BP) were measured. Overweight and obesity were defined according to sex- and age- specific Chinese reference data.

**Results:**

Both SBP and DBP were very significantly increased in overweight (OW) and obese (OB) groups. With age and sex controlled, BMI and WC were independently positively correlated with SBP and DBP. The prevalence of high SBP, DBP and hypertension were markedly higher among OW and OB children than normal weight (NW) group. Odds ratios (ORs) for high SBP, high DBP and high BP were significantly greater in OW and OB children than NW group, and showed a trend increase correlating with obesity stages (all P <0.0001). According to the increasing OR with different combination of obese status of BMI and WC, WC has a stronger influence on hypertension. The combination of BMI and WC obese shows substantially higher ORs compared with those for either BMI or WC obese alone.

**Conclusions:**

In this study on a large school-based population in Shanghai, China, BMI and WC are positively correlated with SBP and DBP. Being overweight or obese greatly increased the risk of hypertension in Chinese children and adolescents, in which WC is considered as a more sensitive indicator than BMI.

## Background

From the recent announcement of National CDC of China, hypertension has become a major public health problem in China. In the 2010 Chinese guidelines for the management of hypertension, the prevalence of hypertension has been increasing in China for decades, and reached 18.8% in the year 2002 [1]. Importantly, these adulthood hypertension can be originally observed to extend back to the childhood. More and more evidence showed hypertensive children are more likely to develop hypertension in adulthood [2-4]. The increase of childhood hypertension not only increases the prevalence of hypertension in later adulthood decades, but also further correlates with increased cardiovascular morbidituy and mortality [5]. Therefore, the study on the prevalence and early diagnosis of hypertension in childhood is an important strategy for the public control and prevention of cardiovascular diseases.

Although the childhood hypertension was once considered to be rare in pediatrics, the ongoing rise in the prevalence of hypertension becomes a common problem in children and adolescents, which is considered to be accompanied with the epidemic of childhood overweight and obesity [6]. Several studies showed that obesity is a well predictor of hypertension [7-9]. However, this correlation of hypertension with obesity in childhood lacks the confirmation from large scale cohort of children and adolescents in China.

Shanghai is the largest city in China, with about 2 million ongoing school children population. Importantly, among them the obese prevalence has greatly increased during the past 20 years [10]. From the statistic data in Shanghai CDC, the obesity prevalence in ongoing children of Shanghai was 3.76% in 1991, but in 2009, the obesity prevalence was increased to be 13.53%, which means now there are more than 200 thousand obese children in Shanghai. Meanwhile, the overweight prevalence was 11.27%. How to take care of these children’s health, especially monitoring the accompanied risk factors, has become an important issue. Therefore, our center established a large scale cross-sectional study, named “Shanghai children and adolescents disease risk factor study” (SCADRFS), in Shanghai from 2009, in which 78,114 children including 40,105 boys and 38,009 girls were observed on their obesity and hypertension stages. Our study present a new evidence for the correlation of hypertension prevalence with overweight and obesity stages in a large population of Chinese children and adolescents.

## Methods

### Study subjects

A school-based cross-sectional study was conducted during the period February 2009 to December 2009 in Shanghai, China. Two-stage cluster sampling technique was used to choose the study sample. A total sample of 78,114 children and adolescents including 40,105 boys and 38,009 girls from the selected schools constituted the subjects of the study. Written informed consent form was obtained from adolescents and their parents. This study was approved by The Ethical Committee of Shanghai CDC.

### Anthropometric measurements

Body weight was determined to the nearest 0.1 kg on standard physician's beam scales with the children and adolescents wearing only the underwear and no shoes. Height was measured to the nearest 0.1 cm on standardized, wall-mounted height boards according to the following protocol: no shoes, heels together, and student's heels, buttocks, shoulders, and head touching the vertical wall surface with line of sight aligned horizontally. Waist circumference (WC) was also measured. Each of the standard physician's beam scales, wall-mounted height boards and body tape measure used to measure were calibrated previously. Body mass index (BMI) was computed by dividing weight (kg) by height squared (m^2^). The age- and gender-specific BMI and WC cutoffs newly developed by the working group on obesity in China (WGOC) were used to define overweight and obesity [11,12]. Overweight is defined by a BMI or WC at or above the 85th percentile and lower than the 95th percentile for children and adolescents of the same age and sex. And obesity is defined by BMI or WC greater than the 95th percentile.

### Blood pressure measurement and high blood pressure definition

Blood pressure was measured by two trained physicians using a standard mercury sphygmomanometer at the right arm with students in the seated position after at least 5 min of rest. The cuff size was based on the length and circumference of the upper arm and was chosen to be as large as possible without having the elbow skin crease obstruct the stethoscope [13]. Blood pressure values were approximated to the nearest 2 mmHg. Systolic blood pressure (SBP) was defined by the first Korotkoff sound (appearance of sounds), and diastolic blood pressure (DBP) was defined by the fourth Korotkoff sound (sound muffling). In order to make the children comfortable in a relaxed environment, measurements were taken in the classroom in the presence of their classmates and teachers without specifying that doctors were performing the activity and doctors wore casual clothes. The age- and gender-specific blood pressure cutoffs in Chinese children and adolescents were used to define pre-hypertension and hypertension [14]. In this definition, hypertension was defined as SBP and/or DBP above the 95th percentile for age and gender.

### Statistical analysis

The continuous variables, including age, weight, height, BMI, and blood pressure, were summarized by the mean and range. Other categorical variables were summarized by count and percentage. Logistical regression model was used to estimate the odds ratios (ORs) and 95% confidence intervals (CIs) between weight status and blood pressure after adjustment for age and gender. Chi-square test was performed to compare the difference between OW and NW, OB and NW. Statistical tests were set with a significance level of 0.05. All data analyses were conducted by using SPSS 15.0 (SPSS Inc., Chicago, Illinois, USA).

## Results

To make an overall enrollment in Shanghai, we studied a large scale of children population covering 110 schools from every district in the city including 60 schools in 9 urban districts and 50 schools in 10 rural districts. As shown in Table 1, totally 78,114 children and adolescents were enrolled for the medical examination, among which 175 cases missing waist circumference data. The average age of the students was around 11 years old (range 7–20 years). Boys in both BMI and WC overweight/obese groups are significantly more than girls (BMI group: OW 16.4% vs.9.7%, OB 12.2% vs.6.6%; WC group: OW 13.0% vs.10.3%, OB 8.5% vs.6.5%). There are more children in urban districts with overweight/obese status than those in rural districts (BMI group: OW 14.6% vs.10.9%, OB 10.8% vs.7.4%; WC group: OW 13.2% vs.9.4%, OB 8.9% vs.5.5%). As shown in Table 1, among groups stratified by BMI and WC for the main anthropometric values, both SBP and DBP were very significantly increased in OW and OB groups, which consistent with previous report that obese children have much higher blood pressure than the normal ones.

**Table 1:** Baseline characteristics of the study population stratified by BMI and WC

	**BMI**				**WC**			
	**Normal weight**	**Overweight**	**Obese**	***P *value**	**Normal weight**	**Overweight**	**Obese**	***P *value**
	**(*n*** **= 60465)**	**(*n*** **= 10252)**	**(*n*** **= 7397)**		**(*n*** **= 62946)**	**(*n*** **= 9109)**	**(*n*** **= 5884)**	
Age (years)	11.3 ± 3.1 (7,20)	11.2 ± 3.0 (7,20)	10.5 ± 2.8 (7,20)	<0.001	11.2 ± 3.1 (7,20)	11.1 ± 2.9 (7,20)	11.5 ± 3.0 (7,20)	<0.001
Sex								
Boys	28652 (71.4%)	6568 (16.4%)	4885 (12.2%)	<0.001	31414 (78.5%)	5210 (13.0%)	3407 (8.5%)	<0.001
Girls	31813 (83.7%)	3684 (9.7%)	2512 (6.6%)		31532 (83.2%)	3899 (10.3%)	2477 (6.5%)	
Residence								
Urban	35067 (74.6%)	6865 (14.6%)	5091 (10.8%)	<0.001	36525 (77.9%)	6184 (13.2%)	4168 (8.9%)	<0.001
Rural	25398 (81.7%)	3387 (10.9%)	2306 (7.4%)		26421 (85.1%)	2925 (9.4%)	1716 (5.5%)	
Height (cm)	147 ± 16 (106,196)	150 ± 15 (110,198)	149 ± 15 (84,196)	<0.001	147 ± 16 (84,196)	150 ± 14 (85,191)	155 ± 15 (117,198)	<0.001
Weight (kg)	38.8 ± 12.5 (13.9,86.7)	50.3 ± 15.3 (18.6,105.5)	57.6 ± 19.1 (17.9,139.1)	<0.001	38.9 ± 12.6 (13.9,101.6)	50.7 ± 13.8 (21.0,105.4)	63.2 ± 18.5 (20.0,139.1)	<0.001
BMI(kg/m^2^)	17.3 ± 2.3 (8.6,30.7)	21.8 ± 2.5 (11.9,27.8)	25.2 ± 3.9 (12.1,63.5)	<0.001	17.5 ± 2.6 (8.6,48.9)	21.9 ± 2.7 (12.1,63.5)	25.7 ± 3.8 (8.7,47.3)	<0.001
WC (cm)	60 ± 7 (26,110)	70 ± 9 (23,101)	78 ± 12 (35,131)	<0.001	60 ± 7 (23,80)	72 ± 6 (58,87)	84 ± 9 (64,131)	<0.001
SBP (mmHg)	102 ± 12 (62,180)	107 ± 13 (70,180)	110 ± 15 (65,180)	<0.001	102 ± 12 (62,180)	107 ± 13 (65,180)	112 ± 14 (70,180)	<0.001
DBP (mmHg)	65 ± 9 (40,137)	67 ± 9 (40,120)	69 ± 10 (40,110)	<0.001	65 ± 9 (40,137)	67 ± 9 (40,120)	70 ± 10 (40,110)	<0.001

Values are expressed as mean ± SD (Min, Max), or *n*(%). P value was calculated by chi-square test for category variable and one-way ANOVA for continuous variable.

BMI, body mass index; WC, waist circumference; DBP, diastolic blood pressure; SBP, systolic blood pressure.

Table 2 made a further analysis on the hypertension prevalence. Classified by BMI or WC, the prevalence of high SBP increased more greatly compared with that of DBP. The prevalence of high SBP, DBP and hypertension were all markedly higher among OW and OB children than NW group. The risk of high BP prevalence was 1.5-2.2 folds in OW and OB children than that in NW group. Odds ratios (ORs) for high SBP, DBP and BP were significantly greater in OW and OB children than NW group, and showed a trend increase correlating with obesity stages(all P <0.0001). BMI and WC were statistically significant after adjusting age and sex.

**Table 2:** Estimated odds ratios and 95% confidence intervals for normal weight, overweight and obese classified by BMI and WC after adjusting for age and sex

	**High SBP *vs *normal SBP**				**High DBP *vs *normal DBP**				**High BP *vs *normal BP**			
	***n *(%)**	**OR**	**95% CI**	***P***	***n *(%)**	**OR**	**95% CI**	***P***	***n *(%)**	**OR**	**95% CI**	***P***
BMI												
NW	3808 (6.30)	1.00			2689 (4.45)	1.00			5243 (8.67)	1.00		
OW	1362 (13.29)	1.67	1.54-1.82	<0.0001	789 (7.70)	1.51	1.36-1.67	<0.0001	1630 (15.90)	1.50	1.39-1.62	<0.0001
OB	1600 (21.63)	2.17	1.94- 2.43	<0.0001	992 (13.41)	2.09	1.83-2.39	<0.0001	1896 (25.63)	1.95	1.76-2.16	<0.0001
WC												
NW	4110 (6.53)	1.00			2894 (4.60)	1.00			5577 (8.86)	1.00		
OW	1261 (13.84)	1.48	1.35-1.62	<0.0001	784 (8.61)	1.35	1.21-1.50	<0.0001	1547 (16.98)	1.47	1.36-1.60	<0.0001
OB	1386 (23.56)	2.25	2.01-2.53	<0.0001	785 (13.34)	1.79	1.56-2.06	<0.0001	1629 (27.69)	2.24	2.02-2.48	<0.0001
Age		1.03	1.02-1.04	<0.0001		0.93	0.92-0.94	<0.0001		1.01	1.00-1.02	0.0255
Sex												
Girls *vs* boys		0.78	0.74-0.82	<0.0001		1.14	1.07-1.21	<0.0001		0.90	0.86-0.94	<0.0001

DBP, diastolic blood pressure; SBP, systolic blood pressure; BMI, body mass index; WC, waist circumference; NW, normal weight; OW, overweight; OB, obese.

Table 3 shows the interaction between different obese status of BMI and WC on the prevalence of high SBP, DBP and BP by the adjusted odds ratio (ORs) with 95% confidence intervals (95% CIs). Controlling for BMI, the adjusted ORs progressively increased with the increasing obese status classified by WC, and vise visa. According to the increasing OR with different combination of obese status of BMI and WC, we can find WC has a stronger influence on high SBP, high DBP and hypertension respectively. For example, when compared with students who were WC overweight, the OR of high BP became higher (1.91 for BMI NW vs. 2.64 for BMI OB, increase 38.2%), while when compared with those who were BMI overweight, the OR of high BP became greater higher with WC obese statues increasing (1.76 for WC NW vs. 2.66 for WC OB, increase 51.1%). Furthermore, the combination of BMI and WC obese shows substantially higher ORs compared with those for either BMI or WC obese alone, 4.66 for BMI OB* WC OB.

**Table 3:** Relationship between different categories of BMI, WC and hypertension after adjusting for age and sex

**Categories**			**High SBP *vs *normal SBP**			**High DBP *vs *normal DBP**			**High BP *vs *normal BP**		
**BMI**	**WC**	***N***	***n *(%)**	**OR(95% CI)**	***P***	***n *(%)**	**OR(95% CI)**	***P***	***n *(%)**	**OR(95% CI)**	***P***
NW	NW	57867	3521 (6.08)	1.00		2509 (4.34)	1.00		4864 (8.41)	1.00	
NW	OW	2211	248 (11.22)	2.00 (1.75, 2.30)	<0.0001	155 (7.01)	1.71 (1.44, 2.02)	<0.0001	326 (14.74)	1.91 (1.69, 2.16)	<0.0001
NW	OB	242	33 (13.64)	2.49 (1.72, 3.61)	<0.0001	19 (7.85)	1.98 (1.23, 3.17)	<0.0001	42 (17.36)	2.32 (1.66, 3.24)	<0.0001
OW	NW	4484	524 (11.69)	1.99 (1.80, 2.19)	<0.0001	335 (7.47)	1.74 (1.54, 1.96)	<0.0001	632 (14.09)	1.76 (1.61,1.92)	<0.0001
OW	OW	4621	645 (13.96)	2.41 (2.20, 2.63)	<0.0001	363 (7.86)	1.95 (1.74, 2.19)	<0.0001	773 (16.73)	2.15 (1.98, 2.34)	<0.0001
OW	OB	1131	190 (16.80)	2.99 (2.55, 3.52)	<0.0001	91 (8.05)	2.18 (1.75, 2.72)	0.0046	224 (19.81)	2.66 (2.29, 3.09)	<0.0001
OB	NW	595	65 (10.92)	1.93 (1.49, 2.51)	<0.0001	50 (8.40)	1.82 (1.36, 2.44)	<0.0001	81 (13.61)	1.72 (1.36, 2.18)	<0.0001
OB	OW	2277	368 (16.16)	2.96 (2.63, 3.33)	<0.0001	266 (11.68)	2.72 (2.37, 3.11)	<0.0001	448 (19.68)	2.64 (2.37, 2.95)	<0.0001
OB	OB	4511	1163 (25.78)	5.22 (4.84, 5.63)	<0.0001	675 (14.96)	3.92 (3.58, 4.30)	<0.0001	1363 (30.22	4.66 (4.34, 5.00)	<0.0001

*N* is the total number in the category. *n* is the number in the category, and% is the proportion in the category with outcome.

Adjusted ORs with 95% confidence intervals (95% CIs) were computed after adjusting for age and sex.

## Discussion

Here we present a cross-sectional, school-based population study covering 110 schools from 19 districts of Shanghai with a large scale of children sample size. We found a significant correlation of children obesity with hypertension prevalence. Our study determined that 22.6% of children were in overweight or obese stage defined by BMI (19.2% defined by WC). Among them, there were significant increase on the prevalence of high SBP, DBP and hypertension. Importantly, the logistic regression analysis showed a positively strong association of obesity stages with the risk of hypertension prevalence in these children.

Being overweight or obese has become highly prevalent in Western countries and is rapidly reaching epidemic proportions in the developing countries. Obesity-related disorders, such as hypertension and diabetes, are also increasing at an alarming rate. In the study from Robert Whitaker et al., obese children under three years of age without obese parents are at low risk for obesity in adulthood, but among older children, obesity is an increasingly important predictor of adult obesity, regardless of whether the parents are obese or not [15].

The criteria for definition of children’s hypertension is arbitrary and to a certain extent, artificial. The normative data for BP in children and adolescents is the United States-based 2004 Task Force Report Update, which has been widely used in recent US-based or European-based studies [16]. Another widely used normative data is the report of WHO expert committee on the Hypertension Control [17]. But whether these standards are applicable to the children and adolescents in the other parts of the world, or the different ethnic backgrounds is unknown, since several studies already showed the difference of children’s BP between children in China and those in western countries [18]. Therefore, in this study, we used Chinese reference standard for children’s hypertension published in 2010, which was established from eleven large scale cross-sectional BP surveys in mainland China from 2001 to 2010, covering four municipalities and seven provinces. Based on the Chinese reference, we made a more suitable evaluation on the Chinese children population and classified the children with hypertension.

Compared with the widely studied on the relation of obesity with hypertension, fewer studies were investigated the prevalence of hypertension in Chinese Children and whether it is related with children’s obesity in China. One recent study conducted Changsha city in China, showed the prevalence of hypertension among adolescents at the age of 12–17 years and found the relation with BMI [19]. Another study also reported the correlation of hypertension with BMI in children population in Hainan province of South China [20]. In this study, we classified the obesity stages of children both by BMI and WC, the two most widely used index for obesity. BMI is most widely used for the indicator of body fatness, but WC indicated the central fat that more likely correlates with diabetes than the association with general fat. Our results showed the overweight and obese groups have apparently higher prevalence of high DBP, high SBP and hypertension, which the obesity stages can be classified both by BMI and WC independently. Interestingly, our data also indicated that WC has a stronger influence on the hypertension, although one group reported that BMI is more sensitive indicator for the prevalence of hypertension in Beijing [21]. Compared with general fat defined by BMI, central obesity defined by WC is the kind of excessive abdominal fat around the stomach and abdomen, which is believed to have a strong association with several health risks, such as type 2 diabetes, asthma and cardiovascular diseases. Especially, it is reported that a central distribution of body fat is associated with increased BP, independently of body mass index and insulin resistance in the middle-aged men [22]. In our study, our finding indicated that in the childhood, the central obesity also has much more stronger association with risk of hypertension compared with general obesity.

## Conclusion

In a conclusion, our large scale school-based study on children population in Shanghai showed the higher prevalence of hypertension was associated with higher BMI or WC overweight and obese children. Being overweight or obese significantly increases the risk of hypertension in the children at the age from 7 to 20 years. Furthermore, the WC seems to be a more sensitive risk factor associated with the risk of hypertension than BMI.

## Competing interest

The author declares to have no proprietary, financial, professional or other interest of any nature or kind in any product, service, and/or company that could be construed as influencing the position presented in this manuscript.

## Authors’ contributions

XL and FW designed the study plan; FW, CYG, CYL, XL, and YFZ organized the school-based population, collected each data; and XL, PS, HTY and FW analyzed the data and wrote the manuscript. All authors read and approved the final manuscript.

## Funding

This work was supported by the National Natural Science Foundation of China (Grant No. 81001250).

## Pre-publication history

The pre-publication history for this paper can be accessed here:

http://www.biomedcentral.com/1471-2458/13/24/prepub
